# *De novo* fatty-acid synthesis and related pathways as molecular targets for cancer therapy

**DOI:** 10.1038/sj.bjc.6605007

**Published:** 2009-04-07

**Authors:** T Mashima, H Seimiya, T Tsuruo

**Affiliations:** 1 Division of Molecular Biotherapy, Cancer Chemotherapy Centre, Japanese Foundation for Cancer Research, 3-10-6 Ariake, Koto-ku, Tokyo 135-8550, Japan; 2 Director's Room, Cancer Chemotherapy Centre, Japanese Foundation for Cancer Research, 3-10-6 Ariake, Koto-ku, Tokyo 135-8550, Japan

**Keywords:** lipid metabolism, fatty-acid synthase, acyl-CoA synthetase

## Abstract

Enhanced lipid biosynthesis is a characteristic feature of cancer. Deregulated lipogenesis plays an important role in tumour cell survival. These observations suggest that enzymes in the lipid synthesis pathway would be rational therapeutic targets for cancer. To this end, we review the enzymes in *de novo* fatty-acid synthesis and related pathways.

To establish an effective strategy in molecular cancer therapeutics, it is essential to identify characteristic features of the disease and to discover ways to selectively target them. Altered metabolism is one of the important features of cancer. Initially, Otto Warburg observed enhanced anaerobic glycolysis in cancer (Warburg, 1956). The elevated glucose catabolism produces an excess of the glycolytic end-product, pyruvate. Most of the pyruvate is converted to lactate, whereas some of it is converted to acetyl-CoA, which, in turn, is used in *de novo* fatty-acid synthesis (Figure 1). Highly proliferating cancer cells need to synthesise fatty acids *de novo* to continually provide lipids for membrane production. The synthesised fatty acids are also used for energy production through *β*-oxidation and lipid modification of proteins. *De novo* fatty-acid synthesis involves two key enzymes, acetyl-CoA carboxylase (ACC) and fatty-acid synthase (FASN). ACC carboxylates acetyl-CoA to form malonyl-CoA. The malonyl-CoA product is further converted by FASN to long-chain fatty acids. Most normal human tissues preferentially use dietary (exogenous) lipid for synthesis of new structural lipids, whereas *de novo* (endogenous) fatty-acid synthesis is usually suppressed, and FASN expression is maintained at low levels. By contrast, in cancer cells, *de novo* fatty-acid synthesis is commonly elevated and the supply of cellular fatty acid is highly dependent on the *de novo* synthesis. Therefore, deregulated *de novo* fatty-acid synthesis directly leads to cellular fatty-acid accumulation and affects fundamental cellular processes, including signal transduction and gene expression. Numerous studies have shown overexpression of FASN in various human epithelial cancers, including prostate, ovary, colon, lung, endometrium and stomach cancers (Kuhajda, 2000). Moreover, several reports have shown that FASN and related lipogenic enzymes play important roles in tumour cell survival at multiple levels.

This minireview summarises recent advances in this field and provides a theoretical basis for the enzymes in lipogenic pathway as a new class of molecular targets to induce cancer selective cell death.

## Regulation of FASN and *de novo* fatty-acid synthesis pathway in cancer

The increased *de novo* fatty-acid synthesis is caused by multiple mechanisms, including increased expression of lipogenic enzymes. Among them, FASN overexpression is observed in a wide variety of human cancers. In cancer cells, transcriptional regulation of *FASN* gene is one of the important mechanisms of FASN overexpression (Kuhajda, 2006; Menendez and Lupu, 2007). The *FASN* expression is regulated by growth factors – growth factor receptors, including epidermal growth factor receptor (EGFR) and HER2, and steroid hormone – steroid hormone receptors, such as oestrogen receptor (ER), androgen receptor (AR) and progesterone receptor (PR). Downstream of the receptors, the phosphatidylinositol-3-kinase (PI3K)-Akt and mitogen-activated protein kinase (MAPK) are candidate signalling pathways that mediate *FASN* expression through the sterol regulatory element-binding protein 1c (SREBP-1c). However, the regulations of *FASN* expression by the growth factor receptors are not simple. For instance, mutual regulation between FASN and the growth factor-dependent signalling is suggested. HER2 induces *FASN* expression through the downstream PI3K signalling (Kumar-Sinha *et al*, 2003). Conversely, FASN-dependent regulation of HER2 expression has also been reported (Menendez *et al*, 2004). Recent reports have further shown that the *FASN* expression is not only controlled by SREBP-1c, but also by other transcription factors, such as the p53 family proteins and the lipogenesis-related nuclear protein, SPOT14, which is overexpressed in breast tumours (D’Erchia *et al*, 2006; Martel *et al*, 2006).

As with these intracellular signalling molecules, extracellular tumour microenvironmental stresses play an important role in tumour-related FASN expression. Solid tumours are commonly characterised by a unique pathophysiological microenvironment, such as hypoxia, low pH, and nutrient starvation. This hostile microenvironment activates several intracellular signalling pathways that promote malignant progression. Hypoxia and low pH stress induce the FASN expression in cancer cells (Menendez *et al*, 2005; Furuta *et al*, 2008). Hypoxia upregulates SREBP-1, the major transcriptional regulator of the *FASN* gene, through phosphorylation of Akt.

**Figure 1:**
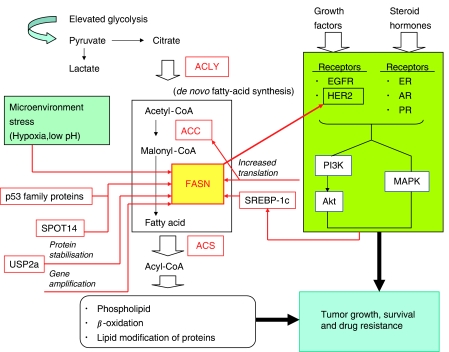
Coupling of elevated fatty-acid metabolism with growth factor signalling in cancer. Growth factors and hormone receptors play essential roles in tumour-related FASN overexpression. FASN and growth factor-dependent signalling are mutually regulated in cancer cells. Tumour microenvironment stress, as well as multiple other factors are involved in FASN overexpression and elevated lipogenesis in cancer. ACLY, ATP citrate lyase; ACC, acetyl-CoA carboxylase; FASN, fatty-acid synthase; ACS, acyl-CoA synthetase; EGFR, epidermal growth factor receptor; ER, oestrogen receptor; AR, androgen receptor; PR, progesterone receptor; PI3K, phosphatidylinositol-3-kinase; MAPK, mitogen-activated protein kinase; SREBP-1c, sterol regulatory element-binding protein 1c; USP2a, ubiquitin-specific protease-2a.

Apart from the transcriptional regulation of FASN, it is also controlled at the posttranslational levels. Graner *et al* showed that the isopeptidase USP2a (ubiquitin-specific protease-2a) interacts with and stabilises FASN protein in prostate cancer (Graner *et al*, 2004). In breast cancer BT-474 cells that overexpress HER2, the expression of FASN and ACC are not mediated by SREBP-1, but by a mammalian target of rapamycin (mTOR)-dependent selective translational induction (Yoon *et al*, 2007). A significant copy number gain of *FASN* gene is also observed in prostate adenocarcinoma (Shah *et al*, 2006). These observations suggest that tumour-related FASN overexpression could be regulated at multiple levels (Figure 1).

## FASN and *de novo* fatty-acid synthesis as promising targets of cancer

FASN is not only overexpressed in cancer, but it also plays an essential role in tumour growth and survival. Two well-known FASN inhibitors, the natural product cerulenin and the synthetic compound c75, have been studied. Treating tumour cells with pharmacological inhibitors of FASN effectively suppresses growth and induces apoptosis in breast cancer cells both *in vitro* and *in vivo* (Pizer *et al*, 2000). Similarly, FASN knockdown by specific siRNAs induces apoptosis in prostate cancer LNCaP cells (De Schrijver *et al*, 2003). By contrast, the RNAi-mediated inhibition of FASN did not affect growth rate or viability of nonmalignant cells. These data indicate that the elevated level of FASN observed in many cancers actually plays a predominant role in tumour growth and survival, suggesting that this enzyme could be a rational therapeutic target.

Although the precise mechanisms of FASN inhibition-induced cell death in cancer cells still remain unknown, several possibilities have been proposed. Initial studies indicate that FASN inhibition accumulates the toxic intermediary metabolite, malonyl-CoA, which induces apoptosis, whereas pharmacological inhibition of ACC by 5-(tetradecyloxy)-2-furoic acid does not (Pizer *et al*, 2000). These studies suggest that malonyl-CoA accumulation, but not end-product fatty-acid depletion, could be a critical factor for apoptosis induction. However, studies with siRNAs revealed that, as with FASN inhibition, ACC inhibition triggers apoptosis in prostate and breast cancer cells, but not in nonmalignant cells (Brusselmans *et al*, 2005; Chajes *et al*, 2006). Inhibiting ACC expression results in a major decrease in the cellular pool of palmitic acid and apoptosis induction. Moreover, supplementing the culture medium with palmitic acid completely rescues cells from both ACC and FASN knockdown-induced apoptosis (Chajes *et al*, 2006). These observations re-emphasise the importance of end-product fatty-acid starvation to cell death. At the same time, these data suggest that, apart from FASN inhibition, ACC inhibition could also be a rational strategy to induce selective death of tumour cells. As described above, FASN and tumour-related survival signals are mutually regulated, and FASN controls HER2-dependent signalling. Besides the role of FASN-dependent fatty-acid synthesis in fatty-acid supply for membrane production and energy production through *β*-oxidation, FASN overexpression is further associated with palmitoylation of signalling proteins (Fiorentino *et al*, 2008). Interference of these pathways could also be involved in the FASN inhibition-induced selective cancer cytotoxicity.

In addition to the essential role of FASN in cancer cell growth and survival, it is involved in other phases of cancer development. Browne *et al* showed that Orlistat, an antiobesity drug, inhibits FASN and suppresses endothelial cell proliferation and angiogenesis, suggesting a novel role of endothelial cell FASN in *in vivo* tumour growth (Browne *et al*, 2006). FASN overexpression also confers resistance to Adriamycin and mitoxantrone in breast cancer cells (Liu *et al*, 2008). These observations suggest that FASN inhibition could be a novel strategy to interfere with tumour survival through angiogenesis and reverse drug resistance of cancer. Recently, it was reported that FASN inhibition induces endoplasmic reticulum stress in cancer cells, and FASN inhibitors cooperate with the endoplasmic reticulum stress inducer to enhance tumour cell death (Little *et al*, 2007). Considering the fact that tumour microenvironment stresses are strong inducers of the endoplasmic reticulum stress (Murakami *et al*, 2007), the FASN inhibitors could show selective and enhanced cytotoxicity to cancer cells under the tumour microenvironment conditions.

The FASN-dependent *de novo* fatty-acid synthesis takes place in cytoplasm. Recently, however, another component of *de novo* fatty-acid synthesis in mitochondria was identified (Hiltunen *et al*, 2009). This suggests that the specificity of FASN inhibitors could be a critical key for successful molecular target therapy of cancer. In this aspect, recent determination of the crystal structure of FASN and molecular description of its active sites (Maier *et al*, 2008) would help in the development of improved inhibitors. Cancer cells also induce lipolysis in adipose tissue, a characteristic feature of cachexia, by producing lipid mobilisation factors (Tisdale, 2004). However, it is still not clear how the exogenous lipid mobilised from adipose tissue could affect the dependence of tumours on *de novo* fatty-acid synthesis. Polymorphisms of *FASN* gene are the other factors that could affect the effectiveness of FASN-targeting agents on cancer (Kovacs *et al*, 2004). Further studies are needed to clarify these points for effective application of the FASN inhibition strategy to cancer therapy.

## Other enzymes in lipogenic pathway as potential cancer targets

### ATP citrate lyase

ATP citrate lyase (ACLY) catalyses the conversion of citrate to cytosolic acetyl-CoA, thus linking the tumour-associated increase in glycolysis to enhanced lipogenesis (Figure 1). As with FASN and ACC, marked elevation of ACLY expression and activity has been reported in cancer cells (Bauer *et al*, 2005; Hatzivassiliou *et al*, 2005). In human lung adenocarcinoma, the expression of phosphorylated ACLY correlated with stage, differentiation grade, and a poorer prognosis (Migita *et al*, 2008). ACLY inhibition by RNAi leads to a significant impairment of glucose-dependent lipid synthesis (Bauer *et al*, 2005). Moreover, ACLY inhibition by siRNAs or the selective inhibitor SB-204990 suppresses the growth and survival of tumour cells *in vitro* and *in vivo* (Bauer *et al*, 2005; Hatzivassiliou *et al*, 2005; Migita *et al*, 2008). Although the molecular mechanisms of ACLY inhibition-dependent cell death are still not clear, these data suggest that this enzyme could also be a target for cancer therapy.

### Acyl-CoA synthetase

Acyl-CoA synthetase (ACS) is an enzyme that acts downstream of FASN and converts long-chain fatty acids to acyl-CoA (Figure 1). This reaction is a critical step in several lipid metabolic pathways, including phospholipid and triacylglycerol biosynthesis, *β*-oxidation, and lipid modification of cellular proteins (reviewed in Coleman *et al*, 2002). In mammals, five ACS isoforms (ACSL1, 3, 4, 5, and 6) have been identified. All the five members catalyse saturated and unsaturated fatty acids of chain lengths 8–22 carbons, whereas ACSL4 prefers arachidonic acid (AA) as a substrate. AA metabolism plays a critical role in colon carcinogenesis. Cao *et al* have shown that intracellular-free AA is a mediator of apoptosis, and ACSL4 suppresses apoptosis by metabolically lowering the intracellular-free AA level (Cao *et al*, 2000). They further showed that ACSL4 is frequently upregulated in colon adenocarcinoma. These observations strongly suggest that ACSL4 could be involved in tumour cell survival. ACSL5, another ACS isoform, plays a role in the pathogenesis of cancer. It is frequently overexpressed in malignant gliomas and is involved in their growth and survival (Yamashita *et al*, 2000; Mashima *et al*, 2009). Studies with a specific inhibitor of ACS, Triacsin c, further revealed that ACS inhibition induces selective apoptosis in cancer cells through the mitochondria-mediated pathway (Mashima *et al*, 2005). These data indicate that ACS is involved in tumour cell survival and that ACS inhibition would be an effective strategy for cancer therapy. At present, it is not clear which ACS member's inhibition would best induce selective tumour cell death. Additional studies are required to clarify the role of each ACS isoform in normal and cancer cells.

## Conclusion

Increased *de novo* fatty-acid synthesis has been recognised as a hallmark of cancer, whereas its significance in cancer pathogenesis has long been underestimated. However, the recent advances in this field have shown the importance of the lipogenic enzymes in tumour cell survival. From the viewpoint of cancer prevention, it would also be important to take into account the effects of dietary factors, such as conjugated linoleic acid, on lipid metabolism in carcinogenesis (Kelley *et al*, 2007). Further investigations on the regulation of these pathways will offer new strategies for tumour treatment, as well as for effective cancer prevention.
